# 

**Published:** 1884-06

**Authors:** 


### MEETING OF DELEGATES.

Delegates to the NATIONAL, and any others who may act in a representative capacity, will meet in Washington City, July 22d, at the Ebbitt House where reduced rates have been made. Reduced railroad rates may be obtained by combination of delegates from the same State or section.

Everything points to a most interesting meeting for organization, enough States have already elected delegates to make the meeting prominent. Favorable responses from every section of the country have shown the time to be most opportune for such a move and the place to be most acceptable.

B. H. CATCHING, *Correspondent*.

ATLANTA, *June 14th*, 1884.

---

### NATIONAL ASSOCIATION OF DENTAL EXAMINERS.

The Second Annual Meeting of the National Association of Dental Examiners will be held at Saratoga Springs, New York, on Friday evening, August 8th, 1884.

All Boards of Examiners are urgently requested to send representatives to this meeting as matters of great importance are expected to be brought before it.

GEO. H. CUSHING, *Sec'y*.

---

### AMERICAN DENTAL ASSOCIATION.

The Forty-Fourth Annual Session of the American Dental Association will be held at Saratoga Springs, New York, commencing at 10 A. M., on Tuesday, August 5th, 1884.

GEO. H. CUSHING, *Rec. Sec'y*.

---

### PENNSYLVANIA STATE DENTAL SOCIETY.

The 16th Annual Session of the Pennsylvania State Dental Society will be held at Wilkers Barre, Pa., July 29th, 1884, and continue in session three days. The meeting will be held at the Wyoming Valley Hotel, which will accomodate three hundred guests. Rates have been reduced from $3.50 to 2.50 per day to delegates, and their families. Excursion rates over the following Rail Roads: The Leheigh Valley will sell to W. B. without special order. The Bloomburg Div., of Del., and Lac. and West, will sell regular excursion tickets from all points on the line. The Philadelphia, Reading and Penna. Central, Northern Central, and Phila. and Erie will sell special tickets over their lines, orders for which (or other information) can be obtained by addressing,

W. H. FUNDENBERG,
*Cor Sec.*

958 Penn Ave., Pittsburgh, Pa.

---

### EDITORIAL, ETC.

### TO READERS AND CORRESPONDENTS.

Communications intended for publication, and exchange journals and papers should be addressed to the EDITOR OF THE AMERICAN JOURNAL OF DENTAL SCIENCE, 259 N. Eutaw St., Baltimore, Md. All letters relating to the business of the JOURNAL, or containing cash remittances, to be directed to Messrs. SNOWDEN & COWMAN. Articles for publication should be received by the editor at least three weeks previous to the first month on which the number in which it is designed for them to appear, is issued.

☞ The AMERICAN JOURNAL OF DENTAL SCIENCE will contain fifty pages of reading matter in each number, making a volume of about *Six Hundred* pages,

EXCLUSIVE OF ADVERTISEMENTS.

THE

### AMERICAN JOURNAL

OF

### DENTAL SCIEN E.

The JOURNAL is issued on the first of each month and contains not less than *fifty* pages of reading matter in each number, exclusive of advertisements, making a volume of *six hundred* pages per annum.

In each number will be found Original Communications, Correspondence, Selected Articles, Monthly Summary, Bibliographical Notices and Editorial Matter, and every effort will be exerted to render the JOURNAL worthy of the patronage of the Dental profession.

A generous co-operation is respectfully solicited from the members of the profession; and as the JOURNAL has now a printing office of its own, which materially lessens the cost of its publication, it will be issued at the reduced subscription price of

**$2.50 Per Annum in Advance.**

Communications are respectfully solicited on all subjects pertaining to practice of dentistry, as well as reports of cases occurring in dental practice.

### RATES OF ADVERTISING.

| | 1 MO. | 2 MO. | 3 MO. | 6 MO. | 12 MO. |
|---|---|---|---|---|---|
| 1 PAGE. | $15 00 | $22 50 | $30 00 | $50 00 | $100 00 |
| ½ " | 10 00 | 15 00 | 20 00 | 30 00 | 50 00 |
| ¼ " | 7 00 | 11 00 | 15 00 | 20 00 | 30 00 |
| ⅛ " | 5 CO | 8 00 | 11 00 | 15 00 | 20 00 |

All original communications designed for publication, works for review, new instruments for notice, exchanges, &c., should be directed to the editor, 259 N. Eutaw St., Baltimore, Md.

All advertisements and subscriptions should be addressed to

SNOWDEN & COWMAN,

*Publishers of the Am., Journal of Dental Science,*

86 W. FAYETTE ST., BET., CHARLES & LIBERTY STS..

BALTIMORE, MD.

